# Research on Assembly Sequence Planning of Large Cruise Ship Cabins Based on Improved Genetic Algorithm

**DOI:** 10.3390/biomimetics10040237

**Published:** 2025-04-11

**Authors:** Liyang Ju, Xiaoyuan Wu, Yixi Zhao, Jianfeng Liu, Kun Liu

**Affiliations:** 1 School of Mechanical Engineering, Shanghai Jiao Tong University, Shanghai 201109, China; yxzhao@sjtu.edu.cn; 2 Shanghai Waigaoqiao Shipbuilding Co., Ltd., Shanghai 200137, China; wxy@chinasws.com (X.W.); liujianfeng@chinasws.com (J.L.); 3 School of Naval Architecture and Ocean Engineering, Jiangsu University of Science and Technology, Zhenjiang 212100, China; kunliu@just.edu.cn

**Keywords:** large cruise ship cabins, assembly sequence planning, genetic greedy combination algorithm, multi-objective optimization

## Abstract

In the construction process of large cruise ships, there are numerous cabin components, and the number of assembly sequences will experience a “combinatorial explosion”, which will become a complex NP hard problem. This article proposes an assembly sequence planning method based on practical engineering problems in the construction process of large cruise ships. The cabin components are modularized, and an optimization algorithm is designed for multi-objective problem solving to obtain the optimal assembly sequence of cabin components. This article analyzes the impact of six constraint conditions on the assembly plan, including geometric constraints, sequence constraints, number of assembly reversals, number of tool replacements, stable connection relationships, and selection of reference components. A fitness function is designed and a mathematical model is established. On this basis, a genetic greedy combination algorithm is proposed to solve the optimal assembly sequence. Compared with traditional genetic algorithms, this improves computational efficiency and solves complex problems in a better manner. Multiple unique optimal solutions can be obtained in one solution process. The feasibility and effectiveness of this method were verified through examples.

## 1. Introduction

The cabins for passengers and crew represent a fundamental unit within the construction of large cruise ships. Typically, shipbuilders employ a modular construction technique, which involves the overall advancement of prefabricated cabin units, to reduce construction costs and enhance the quality of interiors. The construction of these cabin units accounts for approximately one-third of the total building cycle of a large cruise ship. For instance, the domestically produced *Adora Magic City* cruise ship comprises over 1000 modular cabin units. The vast number and variety of component parts involved lead to a phenomenon known as “combinatorial explosion”, which poses a complex NP-hard problem in the sequence planning of cabin assembly. Consequently, optimizing the assembly sequence has been a focal point of interest in cabin construction.

Multiple algorithms exist aimed at optimizing assembly sequences, such as the Genetic Algorithm (GA), Ant Colony Optimization (ACO), and Particle Swarm Optimization (PSO). These algorithms typically converge on a singular optimal solution. In response to complex scenarios, scholars have proposed various specialized optimization techniques. For instance, Su et al. [1] developed a Particle Swarm Optimization algorithm tailored for a hybrid assembly sequence planning issue concerning five assembly parts. Wang et al. [2] introduced a method for assembly sequence planning in discrete manufacturing, achieving an optimal sequence using a Non-dominated Sorting GA with a hybrid chromosome encoding mechanism. Furthermore, Kang et al. [3] explored assembly processes and productivity factors, selecting the sequence that minimized welding deformations based on a simplified analysis, and validated their approach using an asymmetric assembly model. Mingxing et al. [4] proposed an enhanced version of the Ant Colony Algorithm for assembly sequence planning, incorporating factors such as stability, assembly experience, tool changes, and directional changes to construct a more realistically guided evaluation system. Lastly, Chun et al. [5] devised a hybrid algorithm based on the Discrete Particle Swarm, which, after establishing a model for scheduling product assembly tasks, used the Particle Swarm for global searches and Tabu Search to refine the local search capabilities of the algorithm.

Existing research has proposed various optimization algorithms that typically converge to a single optimal result. However, the assembly of large cruise ships is characterized by its complexity and the potential existence of multiple feasible assembly sequences. During the assembly process, various unforeseen circumstances may arise, rendering a single assembly sequence insufficient for addressing these challenges. There is a pressing need to preserve solution diversity among optimal assembly sequences within complex configurations. This study introduces a Genetic Greedy Combination Algorithm (GGCA) tailored to the construction of large cruise ships, aimed at enhancing search efficiency, escaping local optima, achieving the optimal assembly sequence, and producing multiple unique optimal solutions applicable to assembly sequence planning through a single resolution. These solutions are further validated against actual assembly orders.

To address these challenges, we propose a novel Genetic Greedy Combination Algorithm (GGCA) for assembly sequence planning in the construction of large cruise ships. The primary motivation behind this work is to combine the exploration capability of genetic algorithms with a greedy approach to better handle the complexities involved in large-scale ship assembly. Unlike traditional GAs, the GGCA is designed to improve computational efficiency while still providing high-quality solutions. Our approach enables multiple unique optimal solutions to be obtained within a single process, making it particularly suitable for real-world applications where flexibility and efficiency are critical.

The structure of this paper is organized as follows: Section 2 reviews related works and highlights the existing challenges in assembly sequence planning. Section 3 describes the methodology, including the detailed steps of the proposed Genetic Greedy Combination Algorithm (GGCA). The results and discussion, including comparative analyses with other approaches, are presented in Section 4. Finally, Section 5 concludes the paper, providing a summary of findings and potential directions for future research.

By addressing these challenges, our work aims to offer an effective solution to the assembly sequence planning problem, contributing to the advancement of optimization techniques in the shipbuilding industry.

## 2. Problem Description and Model Construction

### 2.1. Problem Description

The installation of cabin units in large cruise ships is a complex engineering task involving multiple steps. In the factory, cabin modules are prefabricated, including walls, floors, ceilings, and built-in furniture, and are then fixed onto the ship’s structure using bolts, welding, and other methods. Connections for electrical systems, plumbing, and ventilation are established to ensure functionality. The installation of furniture, lighting, and carpets completes the interior decoration. The assembly sequence planning issue is a multi-objective optimization problem where the goal is to optimize the modular assembly sequence to avoid problems like the inversion of assembly operations and inefficient repetitive actions. Experimentally validated assembly sequences aim to reduce assembly errors and minimize the risks of unit product errors and interference issues caused by improper sequential relationships. The research approach of this study involves a comprehensive induction of all parts covered by the assembly of modular cabin units, obtaining detailed information on part types, connection methods, installation tools, assembly directions, and sequence constraints. Considerations include factors impacting the total assembly time, such as the number of direction changes, tool replacements, and the selection of reference parts, as well as connection relationships, geometric constraints, and sequence constraints. These factors form the constraints of the problem, which are then solved using the designated algorithm and parameters, with fitness function values compared across identical numbers of iterations. Building on this foundation, all parts are simplified into a segment that conforms to logical assembly constraints. Through iterative constraints and experimental comparisons, this process seeks the optimal assembly sequence for cabin units.

Additionally, the incorporation of modularization in the assembly sequence planning plays a crucial role in optimizing the overall process. Modularization allows for the grouping of components into manageable units, which not only simplifies the installation process but also facilitates better coordination across different teams working on the ship’s construction. By organizing components based on their interdependencies, the assembly process becomes more streamlined, reducing the complexity of handling numerous individual parts. This modular approach also enables parallel work on different sections of the ship, thus accelerating the construction timeline and minimizing potential delays.

Furthermore, the study highlights the importance of real-time adaptability in assembly planning. As the construction process progresses, new challenges may emerge, such as unforeseen structural issues or last-minute design changes. The optimization method employed in this study allows for continuous adjustment of the assembly sequence based on real-time data, ensuring that the plan remains flexible and adaptable to evolving conditions. This adaptability is crucial in a dynamic environment like shipbuilding, where changes are often inevitable. By integrating flexibility into the optimization process, the method ensures that the assembly sequence can be adjusted on the fly without significant disruption to the overall construction schedule.

### 2.2. Model Construction

The establishment of a mathematical model for assembly information is pivotal for the development of assembly sequence planning. This is crucial, as it directly impacts whether the generated assembly sequences are applicable to real-world production challenges [6]. The mathematical model must explicitly represent the product’s part information, assembly tools, and various assembly operations [7].

In the context of assembly sequence planning, it is essential to fully consider the structural and assembly characteristics of the cabin module units. The method involves defining detailed process constraints based on assembly space, order, and worker operational freedom, and then parameterizing these constraints. For the first time, a six-constraint mathematical model has been established, incorporating the mathematical symbols shown in Table 1 to aid in describing the model.

Moreover, the model must also account for factors such as the interactions between different assembly operations, the time required for each operation, and the spatial constraints inherent in the assembly environment. These aspects are vital in ensuring that the planning process is not only efficient but also realistic, aligning with the physical limitations of the workspace and available resources [8]. In addition to considering the fixed constraints, the model also needs to accommodate dynamic factors, such as changes in resource availability, worker skill levels, or unexpected delays. These dynamic elements are captured through adaptive optimization techniques, ensuring that the assembly sequence remains robust and capable of handling variations in real-time production conditions.

Another critical consideration in the development of the assembly sequence planning model is the integration of feedback loops from real-world execution [9]. By incorporating feedback from the actual assembly process, the model can be refined and adjusted over time. This iterative refinement ensures that the planning process evolves alongside the construction process, addressing emerging challenges and improving the accuracy of future sequence plans. Such a feedback-driven approach contributes to the continuous improvement of assembly efficiency, leading to better resource utilization, reduced downtime, and enhanced overall productivity.

➀ Number of Direction Changes

(1) $$F_{1}=\left\{\begin{array}{c} \sum_{i=1}^{N}C_{p,i}+1\;\;\;\;\;\;\;\mathrm{if}\;assembly\;direction\;changes\\ 0\;\;\;\;\;\;\;\;\;\;\;\;\;\;\;\;\;\;\;\;\mathrm{if}\;assembly\;direction\;remains\;unchanged \end{array}\right.$$

In this formula, $C_{p,i}$ represents the number of direction changes required when adjacent assembly parts change their assembly direction. This necessitates additional time and labor costs, thus increasing the number of direction changes. The measure reflects the operational complexity of the assembly sequence. A lower value of $F_{1}$ signifies reduced time costs.

➁ Number of Tool Replacements

(2) $$F_{2}=\left\{\begin{array}{c} \sum_{i=1}^{N}T_{p,i}+1\;\;\;\;\;\;\mathrm{if}\;assembly\;direction\;changes\\ 0\;\;\;\;\;\;\;\;\;\;\;\;\;\;\;\;\;\;\;\;\mathrm{if}\;assembly\;direction\;remains\;unchanged \end{array}\right.$$

Here, $T_{p,i}$ denotes the number of tool replacements when adjacent assembly modules change tools. Using the same assembly tool across modules can enhance efficiency, whereas a change in tools can decrease it. By computing the number of tool replacements, the formula reflects the assembly complexity of the sequence. A lower $F_{2}$ value indicates higher efficiency.

➂ Connection Relationships Among Parts

(3) $$S_{p,i,j}=\left\{\begin{array}{c} \;\;0\;\;\;\;\;\;\;\;\;\;\;\;\mathrm{if}\;\mathrm{there}\;\mathrm{is}\;\mathrm{no}\;\mathrm{contact}\;\mathrm{between}\;P_{i}\;and\;P_{j}\\ 1\;\;\;\;\;\;\;\;\;\;\;\;\mathrm{if}\;\mathrm{there}\;\mathrm{is}\;\mathrm{no}\;\mathrm{contact}between\;P_{i}\;and\;P_{j}\\ \;\;\;\;\;\;\;\;\;\;\;\;\;\;2\;\;\;\;\;\;\;\;\;\;\;\;\mathrm{if}\;\mathrm{there}\;\mathrm{is}\;a\;stable\;connection\;between\;P_{i}\;and\;P_{j} \end{array}\right.$$

(4) $$F_{3}=\sum_{i=2}^{n}S_{p,i,j}$$

Equations (4) and (5) determine the connectivity of the assembly. In the assembly sequence, if adjacent modules $P_{i}$ and $P_{j}$ have no contact, $S_{p,i,j}=0$. If $P_{i}$ and $P_{j}$ are in contact, $S_{p,i,j}=1$. If they are stably connected, such as through welding or bolting, $S_{p,i,j}=2$. The stability of these connections affects the operational reliability and safety of the assembly. A higher value of $F_{4}$ indicates better stability of the assembly sequence, with possible values ranging from $0\leq F_{4}\leq 2N-2$.

➃ Selection of Reference Part

(5) $$B_{L}=\left\{\begin{array}{l} 1\;\;\;\;\;\;\mathrm{if}\;P_{1}\;is\;a\;reference\;part\\ 0\;\;\;\;\;\;\mathrm{if}\;P_{1}\;is\;not\;a\;reference\;part \end{array}\right.$$

In this expression, $B_{L}=1$ if $P_{1}$ is designated as the reference part, otherwise $B_{L}=0$. During the assembly of cabin units, typically one part is fixed and serves as the reference point for assembling all other parts. An assembly containing N parts has N! possible assembly sequences, but specifying a reference part reduces the potential sequences to (N−1)!, thus reducing the search space to 1/N [10] Utilizing a reference part as a heuristic rule greatly diminishes the search space and enhances the efficiency of the generation of assembly sequences. The choice of reference part significantly impacts the difficulty of assembly operations, design of assembly tools, and other factors. Selection criteria for a reference part typically include high connectivity with other parts, substantial weight and low center of gravity, large volume, and high rigidity. If in the initial population a geometrically feasible assembly sequence does not designate $P_{1}$ as the reference part, it becomes necessary to distinguish between feasible sequences with and without $P_{1}$ as the reference [11]. This distinction helps maintain population diversity and weights the fitness of sequences where $P_{1}$ is the reference, enhancing their competitiveness within the population.

➄ Sequential Relationship of Assembly Parts

(6) $$G_{p,i,j}=\left\{\begin{array}{c} \;\;\;\;1\;\;\;\mathrm{if}\;\mathrm{installing}\;P_{j}\;after\;P_{i}\;violates\;geometric\;constraints\\ \;\;\;\;0\;\;\;\mathrm{if}\;installing\;P_{j}\;afterP_{i}\;satisfies\;geometric\;constraints \end{array}\right.$$

(7) $$F_{4}=\sum_{i=1}^{n}G_{p,i,j}$$

Here, if adjacent assembly modules $P_{i}$ and $P_{j}$ interfere with each other, preventing assembly, a penalty coefficient is added to the value of $G_{p,i,j}$, which increases by 1 for each violation. A lower $F_{5}$ value is preferable as it indicates fewer geometric constraint violations.

➅ Geometric Relationships Between Assembly Parts

(8) $$R_{p,i,j}=\left\{\begin{array}{c} \;\;\;0\;\;\;\;\;\;\;\;\mathrm{if}\;\mathrm{installing}\;P_{j}\;after\;P_{i}\;satisfies\;the\;sequence\;constraint\\ \;\;\;1\;\;\;\;\;\;\;\;\mathrm{if}\;\mathrm{installing}\;P_{j}\;after\;P_{i}\;violates\;the\;sequence\;constraint \end{array}\right.$$

(9) $$F_{5}=\sum_{i=1}^{n}R_{p,i,j}$$

In this case, if any part of the sequence between modules $P_{i}$ and $P_{j}$ violates sequence constraints, assembly cannot proceed, indicated by $F_{6}>0$. For example, a sanitary unit in a cabin module cannot be installed before the base trough, contrary to practical installation requirements. A penalty coefficient is applied in the calculation to eliminate sequences that violate these constraints.

### 2.3. Establishment of Fitness Function

Based on the established mathematical model, it is evident that the number of direction changes and the frequency of tool swaps directly impact the efficiency of assembly. The choice of reference components, the stability of connection relationships in the assembly sequence, along with geometric and sequence constraints, determine the feasibility and quality of the assembly sequence. Given that the preparation times for part $P_{i}$, denoted as $O_{p,i}$, $O_{p,t,i}$, and $T_{p,t,i}$, are constants, these are simplified and excluded from the objective function calculations. This leads to the objective function as represented in Equation (11), where $\omega_{1}$, $\omega_{2}$, and${\;\omega }_{3}$ are weighting coefficients satisfying $\omega_{1}+\omega_{2}+\omega_{3}=1$, with values derived from the relative durations required in the actual assembly process: $\omega_{1}=0.2$, $\omega_{2}=0.2$, $\omega_{3}=0.6$, and α=0.8.

(10) $$F_{6}=\frac{1}{\left[\omega_{1}F_{1}+\omega_{2}F_{2}+\omega_{3}\left(2N-2-F_{4}\right)\right]}$$

For sequences where part $P_{1}$ in assembly sequence L is the reference component, the fitness value is adjusted with a weighted coefficient α, less than 1, to enhance the outcome of the fitness function calculation:

(11) $$F_{7}=\frac{1}{\alpha \left[\omega_{1}F_{1}+\omega_{2}F_{2}+\omega_{3}\left(2N-2-F_{4}\right)\right]}$$

If adjacent assembly modules $P_{i}$ and $P_{j}$ interfere with each other due to mutual constraints, a penalty coefficient P, set to 2, is applied in the calculation:

(12) $$F_{8}=\frac{1}{P\;*\;N}$$

Should there be a violation of sequence constraints between neighboring modules $P_{i}$ and $P_{j}$, a larger penalty coefficient μ=8 is implemented to effectively remove problematic sequences:

(13) $$F_{9}=\frac{1}{P\;*\;N\;*\;\mu }$$

In summary, the fitness function is formulated as follows:

(14) $$Fitness\left(L\right)=\left\{\begin{array}{ll} \frac{1}{\left[\omega_{1}F_{1}+\omega_{2}F_{2}+\omega_{3}\left(2N-2-F_{3}\right)\right]} & \;\;\;(a)\\ \frac{1}{\alpha \left[\omega_{1}F_{1}+\omega_{2}F_{2}+\omega_{3}\left(2N-2-F_{3}\right)\right]} & \;\;\;(b)\\ \frac{1}{P\;*\;N} & \;\;\;(c)\\ \frac{1}{P\;*\;N\;*\;\mu } & \;\;\;(d) \end{array}\right.$$

Herein, $Fitness\left(L\right)$ denotes the fitness function value for an assembly sequence. The appropriate formula is selected based on (a) when all constraints are satisfied, (b) when the reference component leads and constraints are met, (c) for geometric constraint violations, and (d) for sequence constraint violations.

## 3. Design of a Genetic Greedy Algorithm

**Figure 1:**
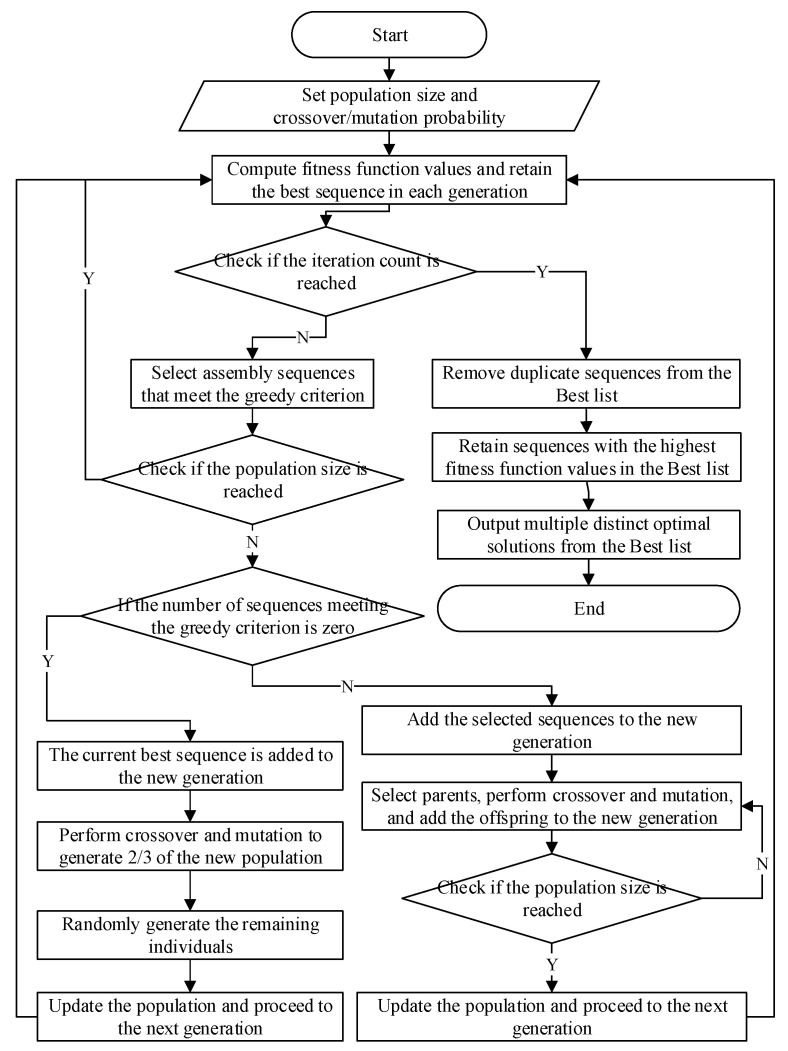
Genetic Greedy Combination Algorithm (GGCA) flowchart.

In response to the issues encountered by the GA when addressing complex problems, such as falling into local optima and converging solely to a singular optimal solution, this study introduces the Genetic Greedy Algorithm (GGA). Compared to the traditional GA, the GGA expands the search space, enabling it to escape local optima and accelerate the convergence process. It is capable of obtaining multiple unique optimal solutions in a single resolution process, thus maximizing its advantages, especially in the context of assembly sequence planning for modular cabin construction in large cruise ships. The algorithmic process is depicted in Figure 1.

Traditional GAs compute the fitness function value for each individual within a population at every generation, comparing these values and retaining the optimal assembly sequence within the population. This method, however, results in the wasteful use of computational resources and reduced computational efficiency. If the population consists of N individuals, the time complexity is O(N). By employing a greedy algorithm to determine the optimal assembly sequence in each generation, only a subset of sequences that meet the greedy criteria need to be evaluated. This approach significantly reduces the computational load and improves efficiency, with a substantial decrease in time complexity. Experimental validation has demonstrated that using GGA for assembly sequence planning is a superior choice.

### 3.1. Algorithm Steps

Following the general concept of the GGA and considering the actual application needs of assembly sequence planning with multiple optimization objectives, the steps of the GGA are as follows:

Step One: Set the initial population size, crossover probability, and mutation probability of the genome. Use random number generation to create the initial population, which serves as the parent population.

Step Two: Analyze the various components of the cabin, connection methods, types of connections, and the assembly tools required. Represent and define the number of direction changes in the assembly, tool change frequency, connections in the assembly, and geometric constraints.

Step Three: Design the fitness function formula based on the constraints and compute the fitness function value for each individual in the population.

Step Four: Retain the assembly sequence with the highest fitness in each generation.

Step Five: Select from the population according to the greedy criteria.

Step Six: Generate a new generation of the population based on the greedy criteria.

Step Seven: Check if the number of iterations has been reached; if so, terminate the loop; if not, proceed to Step Three.

Step Eight: Based on the highest converged fitness function value, eliminate duplicates and sequences that are below this value, retaining only those equal to the highest value.

Step Nine: Output multiple unique optimal solutions.

### 3.2. Greedy Criterion

In this study, constraints inherent to practical engineering problems are employed to establish a greedy criterion. After the GA generates a new generation of the population, this criterion is used to evaluate candidates. If a candidate satisfies the greedy criterion, its fitness function is calculated to select the optimal assembly sequence for that generation. The process for selecting the greedy criterion is as follows:

1. Initially, select an assembly sequence that meets the sequence constraints, such that $F_{5}$ equals 0.
2. On the basis of the first criterion, select an assembly sequence that satisfies geometric constraints, also ensuring that $F_{5}$ equals 0.
3. Continuing from the prior criteria, select an assembly sequence that begins with the reference part, hence $B_{L}$ equals 1.
4. Among the sequences satisfying the first three criteria, select the sequence with fewer direction changes in assembly, aiming for a smaller $F_{1}$ value. A threshold can be set based on the actual conditions of the assembly, and selection occurs if $F_{1}$ is below a certain number of direction changes.
5. From the sequences meeting the first four criteria, choose the sequence with fewer tool replacements, aiming for a smaller $F_{2}$ value. Again, a threshold may be set based on the assembly’s specifics, and selection is made if $F_{2}$ is below a certain number of tool replacements.
6. Among the sequences fulfilling the first five criteria, choose the assembly sequence with the most stable contact relationships, where a greater $F_{4}$ is preferable. A threshold should be set according to the specific conditions of the assembly, with selection occurring if $F_{4}$ exceeds a certain level.

Following the application of the greedy criterion for selection, the chosen assembly sequence represents the individual with the highest fitness function value within the population. This selected individual is then incorporated into the subsequent generation for further evolution. If the quantity of assembly sequences selected using the greedy criterion meets the population requirement for the next generation’s evolution, progression to the next generation occurs immediately. If this requirement is not met, further assessment is necessary, leading to two possible scenarios:

Scenario 1: No assembly sequence in the current generation meets the greedy criterion. To facilitate progression to the next generation, the following steps are undertaken:

1. The optimal assembly sequence identified so far is added to the new generation’s population to ensure that the best individual obtained remains unlost and undamaged by crossover or mutation.
2. The optimal assembly sequence serves as a parent for generating two-thirds of the new population through crossover and mutation, significantly increasing the likelihood of evolving towards superior sequences and augmenting the quantity of high-quality solutions in the new generation.
3. The remaining individuals are generated randomly to expand the search space for the optimal solution, thereby enhancing the probability of escaping local optima.

Scenario 2: Some assembly sequences in the current generation meet the greedy criterion. These sequences are initially added to the new generation’s population. Subsequently, the tournament selection strategy from GA is employed to select parents from these sequences for breeding. Crossover and mutation operations are performed based on predefined probabilities, integrating these into the new generation until the required population size is achieved.

### 3.3. Multiple Unique Optimal Solutions

GAs and other search methods often converge to a single optimal solution, which limits the diversity of outcomes. This limitation becomes particularly pronounced in the assembly of complex parts, where the solution space may contain multiple viable assembly sequences [12]. Should the algorithm identify only one optimal solution, unforeseen changes and emergencies during actual assembly might prevent adherence to the original assembly sequence. The GGCA can obtain multiple unique optimal solutions in a single solution process. The significance of multiple unique optimal solutions is multifaceted: Firstly, in complex assembly scenarios, having multiple optimal assembly sequences provides flexible options to address contingencies, allowing for easy switches to alternative optimal sequences to complete assembly tasks [13]. Secondly, in the context of real-world engineering problems, multiple unique optimal solutions offer a more accurate and practical reflection of the complexity and uncertainty in actual assembly situations, thus providing more precise and viable solutions [14]. Overall, by providing multiple optimal solutions, the GGCA effectively overcomes the limitations of traditional GAs and other search algorithms in complex assembly issues, thereby enhancing the algorithm’s robustness and adaptability to real-world assembly challenges.

## 4. Genetic Greedy Combination Algorithm: An Overview

The GGCA is implemented through a series of steps that begin with the genetic encoding of components within the passenger cabins of large cruise ships using a genome-based encoding method derived from GA. This is followed by crossover and mutation operations, and then selection using a greedy criterion. Upon completion of the selection process, multiple unique optimal solutions are established and, upon termination of iterations, the computation concludes.

### 4.1. Genome-Based Encoding Method

The method of transforming a problem’s candidate solutions from their solution space into the search space manageable by GA is referred to as encoding [15]. Common encoding techniques include binary encoding, real number encoding, and permutation encoding [16]. This study employs decimal real number encoding, where each numerical code represents a corresponding cabin component. If a cabin has NNN components, then the length of the encoding is N.

#### 4.1.1. Crossover

The method adopted for chromosome crossover in this study involves randomly selecting two parental chromosomes, A and B, and performing a crossover operation between them. A crossover region, arbitrarily chosen (e.g., positions 5 to 9), is selected within the chromosomes. In this region, the genetic material from both parents is exchanged to create two offspring [17]. Specifically, the genes from chromosome A are inherited by one offspring, while the genes from chromosome B are inherited by the other. This process simulates natural reproduction, where genetic traits are shared and recombined in different ways to produce diversity in the population.

The crossover operation begins by selecting the positions within the chromosomes at which the crossover will occur [18]. This is typically done by generating random crossover points, and the chosen positions are used to define the boundaries of the crossover region. Once the region is identified, the genetic sequence from parent A is swapped with that from parent B within this region. For example, if the crossover occurs between positions 5 and 9, the genes at positions 5–9 in chromosome A are exchanged with those of chromosome B. The remaining genes outside this region (positions 1–4 and 10 onwards) are retained from their respective parents.

This approach ensures that the offspring inherit a combination of genetic information from both parents, potentially preserving beneficial traits while allowing for the exploration of new combinations of genes. The diversity generated by this crossover operation plays a crucial role in the evolution of the population, increasing the likelihood of discovering optimal or near-optimal solutions over successive generations. Additionally, the crossover operation is designed to be performed with a certain probability (crossover rate), which governs how frequently it occurs during the algorithm’s execution [19]. This introduces a controlled level of variation, which is essential for balancing exploration (searching through the solution space) and exploitation (refining the best solutions found).

By implementing such a crossover technique, the algorithm promotes genetic diversity, which can improve the robustness and effectiveness of the genetic search process, particularly in complex problems, such as assembly sequence planning. The crossover operation is critical in achieving a successful balance between maintaining high-quality solutions and exploring new potential solutions.
Parent A =123456789101112131415Parent B =324191411155107121368

The segments from A (5, 6, 7, 8, 9) and B (9, 12, 11, 15, 5) undergo crossover, resulting in the following:
Parent A =12345678991411155101112131415Parent B =32419141115556789107121368

Subsequent removal of duplicate components from A and B yields offspring C and D:
Offspring C =123456789141115101213Offspring D =324191411155678101213

#### 4.1.2. Mutation

The chromosome mutation process in this study involves selecting two chromosomes, A and B, for mutation. Assuming a randomly chosen mutation position is between 4 and 9 [18].
Parent A =123456789101112131415Parent B =324191411155107121368

Mutation involves reversing the positions in A (4, 5, 6, 7, 8, 9 to 9, 8, 7, 6, 5, 4) and in B (1, 9, 14, 11, 15, 5 to 5, 15, 11, 14, 9, 1), resulting in mutated offspring:
Offspring C =123987654101112131415Offspring D =324515111491107121368

### 4.2. Establishment of Multiple Unique Optimal Solutions

**Figure 2:**
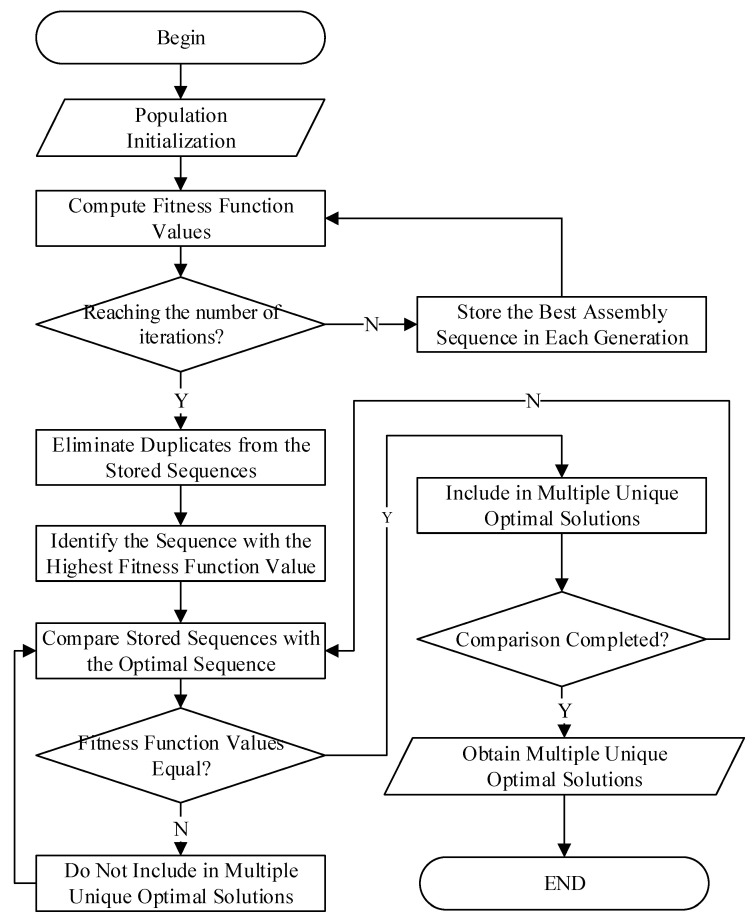
Process flow for determining multiple unique optimal solutions.

The establishment of multiple unique optimal solutions does not involve merely executing the algorithm multiple times to obtain several solutions. Repeated execution of the algorithm is likely to yield the same optimal assembly sequence, thus failing to achieve uniqueness in the solutions and resulting in significant wastage of computational resources. The method employed in this study involves preserving the assembly sequence with the highest fitness at the end of each generation’s selection process. Assuming that the number of iterations is denoted by $P_{n}$, $P_{n}$ optimal assembly sequences are retained. Upon completion of the iterations, duplicate assembly sequences are removed from the stored optimal sequences to maintain their uniqueness. Subsequently, sequences with fitness values below the convergent highest fitness are discarded. The remaining assembly sequences constitute the multiple unique optimal solutions. The process is illustrated in Figure 2.

This approach ensures that the algorithm does not merely converge to a single solution but instead explores a diverse set of high-quality solutions, each representing a potentially different optimal strategy for assembly planning. By removing duplicates, the algorithm guarantees that the final set of solutions contains genuinely distinct assembly sequences, preventing redundancy and fostering greater exploration of the solution space. The uniqueness of the optimal sequences is particularly important in complex, real-world applications, such as large cruise ship assembly, where multiple feasible approaches may exist to achieve the same performance, but with varying trade-offs in time, cost, or resource usage.

To further enhance the process, the fitness values of the retained solutions are used to establish a ranking system. This ranking allows the most efficient assembly sequences to be identified and prioritized in future generations. The diversity among the retained sequences is maintained by evaluating their fitness against the highest convergent fitness value, ensuring that only the best solutions, which exhibit distinct assembly paths, are considered. This method also helps avoid the premature convergence often observed in traditional genetic algorithms, where the population may lose diversity early on and get stuck in local optima.

In addition to this, the algorithm’s efficiency is enhanced by utilizing a diversity preservation strategy, which includes checking for similarity between the newly generated solutions and previously stored ones. This prevents the population from being dominated by a small set of similar solutions and maintains the diversity needed for a more comprehensive search through the solution space. The process, illustrated in Figure 2, is designed to ensure that each generation contributes to the discovery of new and unique assembly sequences, thus improving the likelihood of finding the optimal solution for each unique constraint set encountered in the ship assembly process.

Furthermore, as the algorithm proceeds through the generations, the solutions retained in each iteration provide valuable insights into the evolution of the assembly sequence and the impact of various constraints on the final outcome. This allows for adaptive optimization, where the system can adjust its search focus based on the performance of previous iterations, ultimately leading to a set of assembly sequences that are not only optimal but also adaptable to changing conditions and requirements.

## 5. Case Study Validation

### 5.1. Case Verification

**Figure 3:**
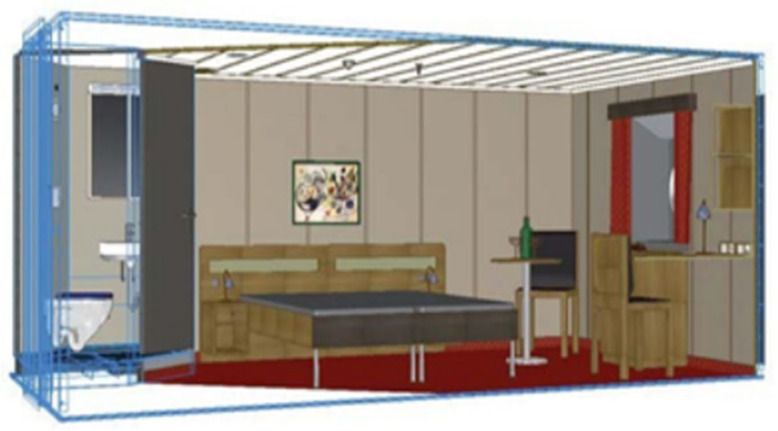
Modular cabin of a large cruise ship.

Experiments were conducted within a compartment of a shipyard, specifically a cabin unit primarily comprising sanitary units, wall panel systems, ceiling systems, fire doors, furniture systems, ventilation systems, sprinkler systems, and electrical systems. A schematic of the assembled cabin is shown in Figure 3.

The connections between components primarily involve welding, bolting, riveting, and adhesive bonding. The assembly of the cabin module requires tools such as cutting machines, welding machines, electric drills, jigsaws, diagonal pliers for electricians, crimping pliers, flat and Phillips screwdrivers, voltage testers, cable ties, aluminum ladders, crowbars, and laser levels. A 20-m chalk line is also used. Table 2 describes the simplified cabin module components, assigning numbers to them and defining their connection methods, installation tools, assembly directions, whether they are reference components, and sequence constraint relationships.

To ensure reliable experimental results, the parameters for two different algorithms were set to identical values. The population size was set at 200, with a crossover rate of 0.3 and a mutation rate of 0.1. Geometric constraints, sequence constraints, number of direction changes, number of tool replacements, stable connection relationships, and reference part conditions were all uniformly applied, and the same fitness function evaluation strategy was used. The GA employed a tournament selection method, with experiments conducted over 300 and 600 generations.

**Figure 4:**
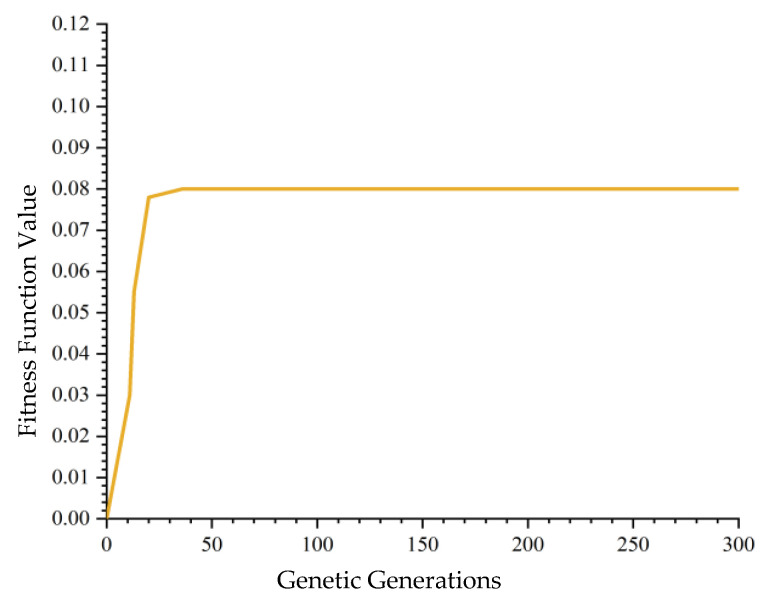
GA convergence plot with 300 iterations.

For a set number of 300 generations, the GA was executed ten times, with the highest fitness value recorded at 0.1157. The results, as shown in Table 3. Figure 4 selects one of the convergence graphs for observation. indicate that the GA frequently became trapped in local optima, suggesting its inadequacy for addressing the assembly challenges of large cruise ship modular cabins. This necessitates further improvements to the algorithm.

**Figure 5:**
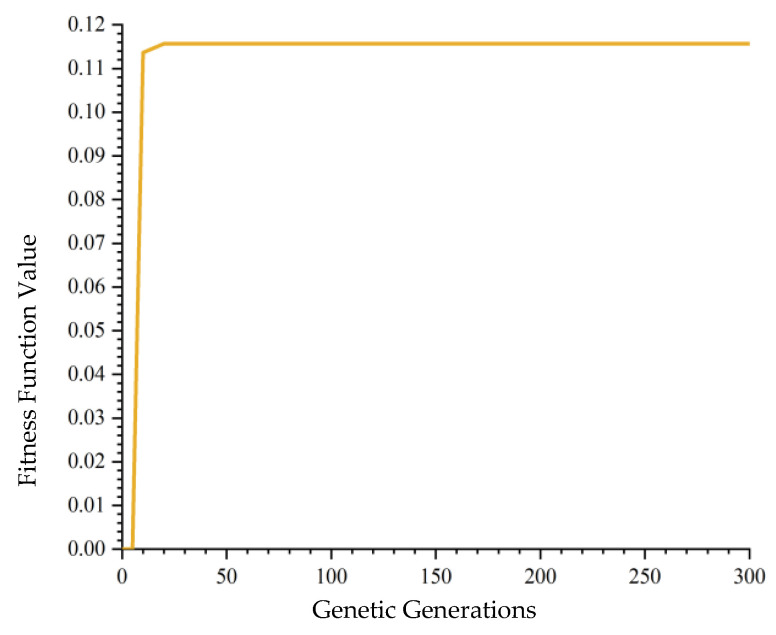
GGCA convergence graph with 300 iterations.

**Figure 6:**
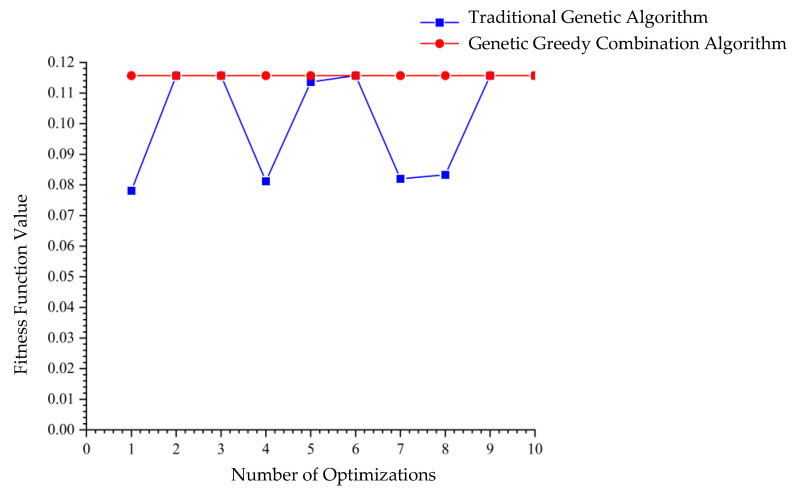
Comparison of fitness function value changes with 300 iterations.

A single execution of the GGCA yielded ten unique optimal solutions, as shown in Table 4. Figure 5, which illustrates the convergence plot of the algorithm, demonstrates that GGCA converges to the optimal value in fewer than 20 generations, exhibiting rapid convergence, high efficiency, and a reduced propensity for entrapment in local optima. Figure 6 compares the fitness function value changes across ten runs of the GA with a single run of GGCA. GA achieved optimal results in only four out of ten trials, with the remaining six trials resulting in local optima. In contrast, a single run of GGCA produced ten unique optimal solutions. Both in terms of convergence speed and the number of optimal solutions obtained, GGCA significantly outperforms GA.

**Figure 7:**
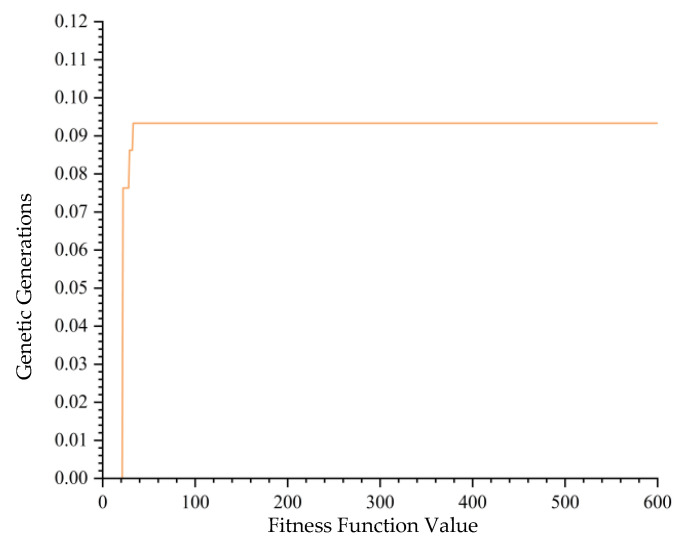
GA Convergence Graph with 600 Iterations.

When the iteration count was set to 600, ten runs using GA achieved a highest fitness function value of 0.1157, as outlined in Table 5. Among these ten runs, only three successfully identified the optimal assembly sequences, while the remainder became stuck in local optima. Figure 7, which selects one of these convergence plots for examination, clearly shows the algorithm’s entrapment in a local optimum. This suggests that GA is still inadequate for addressing the assembly sequence planning of modular cabin units in large cruise ships when run for 600 iterations.

**Figure 8:**
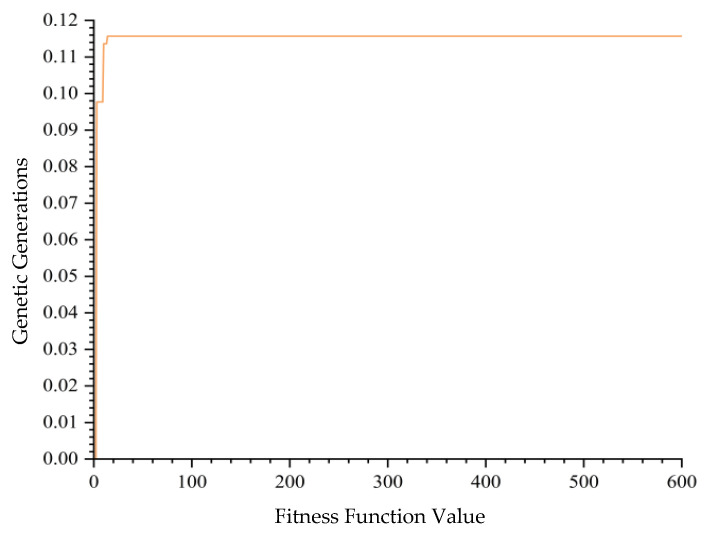
GGCA convergence graph with 600 iterations.

**Figure 9:**
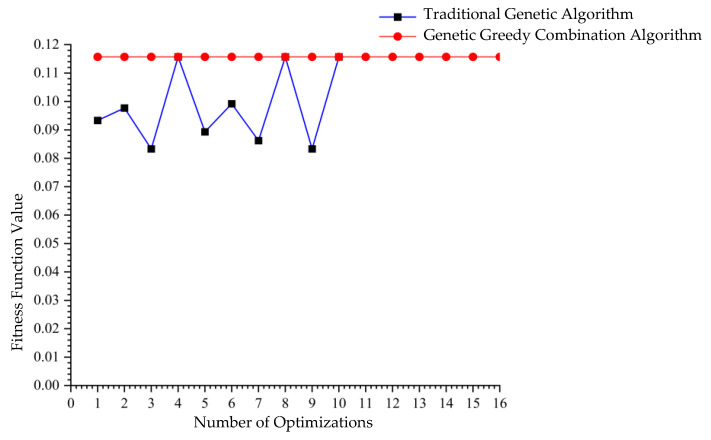
Comparison of fitness function value changes with 600 iterations.

Conversely, a single run of GGCA produced multiple unique optimal solutions, as detailed in Table 6, identifying sixteen unique optimal outcomes with the highest fitness function value also at 0.1157. Figure 8 shows the convergence plot for GGCA, indicating a faster and more stable convergence rate compared to GA. Figure 9 compares the changes in fitness function values across ten runs of GA and a single run of GGCA, where GA reached the optimal solution in only three out of ten instances, succumbing to local optima in the remaining cases. Meanwhile, GGCA, in just one run, successfully discovered sixteen unique optimal solutions. Whether considering the speed of convergence or the number of optimal solutions, the GGCA algorithm markedly surpasses GA, proving more suitable for solving the assembly problems of modular cabin units on large cruise ships.

### 5.2. Analysis and Comparison of Experimental Results

The experimental outcomes indicate that the Genetic Algorithm (GA) exhibits instability in convergence, particularly at 300 and 600 iterations, where it frequently becomes trapped in local optima. A closer examination of the convergence trajectory reveals that the GA tends to settle into local optima early on in the optimization process, and simply increasing the number of iterations does not significantly improve the situation. This behavior is primarily due to the increased complexity of the problem, the larger number of constraints, and the expanded search space. As the GA progresses, the fitness value of the solutions becomes high, and finding individuals with superior fitness in the increasingly sparse search space becomes more difficult. This results in a stagnation of the optimization process, hindering further exploration of potentially better solutions.

Furthermore, as the population evolves over successive generations, it tends to become more homogeneous, diminishing diversity within the population. The reduction in diversity significantly impairs the algorithm’s ability to escape local optima, as the mutation process becomes less effective at introducing new, potentially better solutions. Consequently, the GA struggles to make meaningful progress in complex problems, making it clear that further enhancements are required to improve its performance, especially in the context of practical engineering applications where optimal solutions are crucial.

In contrast, the Genetic Greedy Combination Algorithm (GGCA) proposed in this study effectively overcomes the limitations of the traditional GA. Unlike the GA, GGCA is capable of achieving multiple unique optimal solutions in a single optimization run. By deeply integrating the greedy algorithm with the GA, the GGCA enhances the overall search efficiency. The greedy algorithm’s ability to quickly focus on promising solutions complements the GA’s global search capability, leading to a more balanced and efficient optimization process.

Experimental results demonstrate that GGCA consistently produces a greater number of optimal solutions and achieves faster convergence compared to the traditional GA. Regardless of the iteration count, GGCA not only outperforms the GA in terms of the number of high-quality solutions found but also exhibits more reliable and stable convergence. This makes GGCA particularly well-suited for addressing the complex problem of assembly sequence planning for large cruise ship modular cabin units. The ability to generate multiple optimal solutions offers significant flexibility, ensuring that the assembly process can be adapted to varying constraints and environmental conditions.

GGCA proposed in this study provides a more effective approach to solving the assembly sequence planning problem, addressing the shortcomings of the GA and offering a more efficient and robust solution. This work provides valuable insights and practical guidance for the planning and optimization of modular assembly processes, particularly in large-scale and complex industrial applications like the construction of cruise ships.

## 6. Conclusions

Considering the diversity in the number and structure of cabin units on large cruise ships, this study introduces the Genetic Greedy Combination Algorithm (GGCA) for assembly sequence planning of modular cabin units. This approach is designed to address the complexities inherent in the assembly process, where each unit may vary in size, shape, and structural requirements. A comprehensive mathematical model incorporating six key constraints is developed to accurately capture these complexities. The model is then solved using a multi-objective optimization approach, which leverages the strengths of the hybrid algorithm, combining the global search capabilities of the genetic algorithm with the local search efficiency of the greedy algorithm.

The integration of these two algorithms is particularly effective in enhancing the genetic algorithm’s ability to search large, complex solution spaces. The global search capability of the genetic algorithm ensures that the solution space is thoroughly explored, preventing the algorithm from getting trapped in local optima. Meanwhile, the local search efficiency of the greedy algorithm allows for quicker convergence within promising areas of the solution space, significantly improving the overall search efficiency. This synergy between the two algorithms facilitates the identification of global optimal solutions, even in challenging, high-dimensional environments where traditional optimization methods might struggle.

The simulation results validate the effectiveness of the hybrid algorithm, demonstrating that it successfully mitigates the common issue of genetic algorithms converging prematurely to local or suboptimal solutions. By generating multiple optimal assembly sequences that satisfy all the specified constraints, the hybrid algorithm offers a range of viable solutions for use in onsite assembly planning. These solutions are not only optimal but also adaptable to changes in the assembly process, providing flexibility in response to dynamic conditions on the production floor.

Moreover, one of the key advantages of this method is its ability to generate multiple viable assembly sequences within a single optimization process. This is particularly useful in real-world scenarios where flexibility is crucial, such as in the presence of external environmental changes or unexpected disruptions in the assembly workflow. These multiple solutions serve as effective contingency plans, ensuring that the assembly process can continue smoothly even when unforeseen challenges arise. In this context, the algorithm provides a robust tool for shipbuilders to optimize their assembly processes, reduce costs, and improve efficiency while maintaining the flexibility to adapt to changing conditions.

## Figures and Tables

**Table 1:** Definition of mathematical symbols.

Symbol	Definition
N	Number of parts in the product
L	Assembly sequence for the cabin
$P_{i}$	The i-th assembly part in the assembly sequence
$O_{p,i}$	Setup time for part $P_{i}$ in the assembly sequence
$O_{p,t,i}$	Operation time for part $P_{i}$ in the assembly sequence
$C_{p,i}$	Number of changes in assembly direction for part $P_{i}$
$C_{p,t,i}$	Additional work time due to a change in assembly direction for part $P_{i}$
$T_{p,i}$	Number of tool changes during the assembly of part $P_{i}$
$T_{p,t,i}$	Additional work time due to a tool change for part
$S_{p,i,j}$	Connection relationship between parts $P_{i}$ and $P_{j}$
$B_{L}$	Whether part $P_{1}$ in the assembly sequence is a reference part
$G_{p,i,j}$	Whether parts $P_{i}$ and $P_{j}$ satisfy geometric constraints
$R_{p,i,j}$	Whether parts $P_{i}$ and $P_{j}$ satisfy sequence constraints

**Table 2:** Part constraint information.

Component Name	ID	Connection Method	Installation Tool/ID	Assembly Direction	Reference Component	Sequential Constraint
Cabin Base Groove	1	Welding	Welding Machine T1	X	Yes	None
Sanitary Unit	2	Welding	Welding Machine T1	X	No	1–2
Cabin Wall Panel	3	Self-tapping Screws	Electric Drill T2	Y	No	2–3
Fireproof Door Frame	4	Welded Bolt Connection	Welding Machine T1	X	No	None
Cabin Ceiling	5	Anchor Connection	Screwdriver T3	Y	Yes	3–5
Temporary Reinforcement	6	Bolts	Electric Drill T2	−X	No	None
Furniture	7	Bolts	Electric Drill T2	−X	No	None
Ceiling Trim Strip	8	Bolts	Electric Drill T2	−Y	No	None
Permanent Reinforcement	9	Bolts	Electric Drill T2	Y	No	None
Water Pipes and Sprinkler Heads	10	Bolts	Electric Drill T2	Y	No	5–10
Electrical Equipment	11	Bolts	Electric Drill T2	Y	No	None
Switches and Sockets	12	Bolts	Electric Drill T2	Z	No	11–12
Fireproof Door	13	Bolts	Electric Drill T2	Z	No	4–13
Cabin Unit Protection	14	Bolts	Electric Drill T2	−Z	No	None
Cable	15	Anchor Connection	Screwdriver T3	−Z	No	3–15

**Table 3:** Results of 10 GA runs over 300 iterations.

Iterations	Optimal Assembly Sequence	Fitness Function Value	Number of Part Direction Changes	Number of Tool Changes	Connection Stability	$P_{1}$ as Reference Component	Satisfies Sequence Constraints	Satisfies Geometric Constraints
1	1,6,4,13,2,3,8,14,15,5,9,11,10,12,7	0.0781	10	7	7	Yes	Yes	Yes
2	1,4,2,8,11,9,3,5,15,14,7,6,12,13,10	0.1157	6	3	13	Yes	Yes	Yes
3	1,4,2,3,5,11,10,9,8,13,12,7,6,14,15	0.1157	5	4	13	Yes	Yes	Yes
4	1,11,14,2,3,5,4,9,10,8,7,6,12,13,15	0.0812	10	7	8	Yes	Yes	Yes
5	1,2,4,9,3,15,5,11,10,8,6,7,12,13,14	0.1136	7	3	13	Yes	Yes	Yes
6	1,4,2,8,3,5,11,10,9,7,6,13,12,14,15	0.1157	5	4	13	Yes	Yes	Yes
7	9,1,2,4,14,11,8,3,5,10,6,7,13,12,15	0.0820	8	5	12	No	Yes	Yes
8	11,8,12,1,4,2,9,3,5,10,13,7,6,14,15	0.0833	7	5	12	No	Yes	Yes
9	1,4,2,8,7,6,3,9,11,5,10,13,12,14,15	0.1157	5	4	13	Yes	Yes	Yes
10	1,4,2,8,3,5,15,14,6,7,9,11,10,12,13	0.1157	6	3	13	Yes	Yes	Yes

**Table 4:** Results of one GGCA run with 300 iterations.

Iterations	Optimal Assembly Sequence	Fitness Function Value	Number of Part Direction Changes	Number of Tool Changes	Connection Stability	$P_{1}$ as Reference Component	Satisfies Sequence Constraints	Satisfies Geometric Constraints
1	1,4,2,3,5,15,14,9,10,11,8,13,12,7,6	0.1157	6	3	13	Yes	Yes	Yes
2	1,2,4,9,3,5,10,11,8,12,13,6,7,14,15	0.1157	5	4	13	Yes	Yes	Yes
3	1,4,2,3,9,11,12,13,7,6,8,14,15,5,10	0.1157	6	3	13	Yes	Yes	Yes
4	1,4,2,3,5,15,14,11,9,10,8,6,7,13,12	0.1157	6	3	13	Yes	Yes	Yes
5	1,2,4,7,6,3,5,15,14,8,11,9,10,13,12	0.1157	6	3	13	Yes	Yes	Yes
6	1,4,2,7,6,8,3,14,15,5,9,11,10,13,12	0.1157	6	3	13	Yes	Yes	Yes
7	1,2,4,8,7,6,3,9,5,15,14,11,10,12,13	0.1157	6	3	13	Yes	Yes	Yes
8	1,2,4,8,3,14,15,5,9,10,11,6,7,13,12	0.1157	6	3	13	Yes	Yes	Yes
9	1,4,2,7,6,11,9,3,8,14,15,5,10,12,13	0.1157	6	3	13	Yes	Yes	Yes
10	1,2,4,8,6,7,11,9,3,5,10,12,13,14,15	0.1157	5	4	13	Yes	Yes	Yes

**Table 5:** Results of 10 GA Runs with 600 Iterations.

Iterations	Optimal Assembly Sequence	Fitness Function Value	Number of Part Direction Changes	Number of Tool Changes	Connection Stability	$P_{1}$ as Reference Component	Satisfies Sequence Constraints	Satisfies Geometric Constraints
1	1,11,8,4,2,3,5,15,14,13,12,9,10,6,7	0.0933	8	5	10	Yes	Yes	Yes
2	1,2,7,3,5,4,8,9,11,10,13,12,6,14,15	0.0977	8	5	11	Yes	Yes	Yes
3	11,6,1,4,2,12,13,9,3,7,8,14,15,5,10	0.0833	8	4	12	No	Yes	Yes
4	1,2,4,8,6,7,3,5,15,14,10,9,11,12,13	0.1157	6	3	13	Yes	Yes	Yes
5	4,1,2,14,11,13,12,7,6,8,9,3,5,15,10	0.0893	7	3	13	No	Yes	Yes
6	1,2,3,8,14,15,5,4,9,10,11,7,6,13,12	0.0992	8	4	11	Yes	Yes	Yes
7	8,1,2,4,7,6,3,9,5,15,14,10,11,12,13	0.0862	6	4	12	No	Yes	Yes
8	1,2,4,7,6,3,5,11,10,9,8,12,13,14,15	0.1157	5	4	13	Yes	Yes	Yes
9	11,14,4,1,2,8,6,7,3,13,12,15,5,10,9	0.0833	8	4	12	No	Yes	Yes
10	1,2,4,8,3,9,11,5,15,14,10,13,12,7,6	0.1157	6	3	13	Yes	Yes	Yes

**Table 6:** Results of one GGCA run with 600 iterations.

Iterations	Optimal Assembly Sequence	Fitness Function Value	Number of Part Direction Changes	Number of Tool Changes	Connection Stability	$P_{1}$ as Reference Component	Satisfies Sequence Constraints	Satisfies Geometric Constraints
1	1,2,4,8,3,5,11,10,9,13,12,6,7,14,15	0.1157	5	4	13	Yes	Yes	Yes
2	1,4,2,8,9,3,5,15,14,7,6,11,10,12,13	0.1157	6	3	13	Yes	Yes	Yes
3	1,4,2,3,5,11,10,9,12,13,6,7,8,14,15	0.1157	5	4	13	Yes	Yes	Yes
4	1,2,4,6,7,8,9,3,5,15,14,10,11,13,12	0.1157	6	3	13	Yes	Yes	Yes
5	1,4,2,6,7,3,9,11,5,15,14,8,10,12,13	0.1157	6	3	13	Yes	Yes	Yes
6	1,4,2,8,6,7,3,9,11,14,15,5,10,13,12	0.1157	6	3	13	Yes	Yes	Yes
7	1,4,2,8,3,5,15,14,11,9,10,7,6,13,12	0.1157	6	3	13	Yes	Yes	Yes
8	1,2,4,6,7,11,9,3,14,15,5,10,8,13,12	0.1157	6	3	13	Yes	Yes	Yes
9	1,2,4,9,3,5,15,14,6,7,11,10,8,12,13	0.1157	6	3	13	Yes	Yes	Yes
10	1,4,2,3,5,11,10,9,6,7,8,13,12,14,15	0.1157	5	4	13	Yes	Yes	Yes
11	1,2,4,7,6,8,9,3,5,15,14,11,10,13,12	0.1157	6	3	13	Yes	Yes	Yes
12	1,2,4,8,11,9,3,5,15,14,6,7,10,12,13	0.1157	6	3	13	Yes	Yes	Yes
13	1,4,2,3,14,15,5,11,9,10,12,13,8,7,6	0.1157	6	3	13	Yes	Yes	Yes
14	1,4,2,7,6,8,3,5,15,14,10,9,11,13,12	0.1157	6	3	13	Yes	Yes	Yes
15	1,4,2,3,9,14,15,5,11,10,12,13,8,6,7	0.1157	6	3	13	Yes	Yes	Yes
16	1,2,4,7,6,3,5,15,14,10,9,11,13,12,8	0.1157	6	3	13	Yes	Yes	Yes

## Data Availability

The data presented in this study are available on request from the corresponding author.
